# A simple method for isolation and construction of markerless cyanobacterial mutants defective in acyl-acyl carrier protein synthetase

**DOI:** 10.1007/s00253-016-7850-8

**Published:** 2016-10-04

**Authors:** Kouji Kojima, Sumie Keta, Kazuma Uesaka, Akihiro Kato, Nobuyuki Takatani, Kunio Ihara, Tatsuo Omata, Makiko Aichi

**Affiliations:** 1 Department of Biological Chemistry, Chubu University, Kasugai, 487-8501 Japan; 2 Japan Science and Technology Agency, CREST, Kawaguchi, Japan; 3 Graduate School of Bioagricultural Sciences, Nagoya University, Nagoya, 464-8601 Japan; 4 Graduate School of Bioagricultural Sciences, Nagoya University, Nagoya, 464-8601 Japan; 5 Center for Gene Research, Nagoya University, Nagoya, 464-8602 Japan

**Keywords:** Acyl-ACP synthetase, Biofuel, Cyanobacteria, Free-fatty acid, Counter selection, Sensitivity to FFAs

## Abstract

Cyanobacterial mutants defective in acyl-acyl carrier protein synthetase (Aas) secrete free fatty acids (FFAs) into the external medium and hence have been used for the studies aimed at photosynthetic production of biofuels. While the wild-type strain of *Synechocystis* sp. PCC 6803 is highly sensitive to exogenously added linolenic acid, mutants defective in the *aas* gene are known to be resistant to the externally provided fatty acid. In this study, the wild-type *Synechocystis* cells were shown to be sensitive to lauric, oleic, and linoleic acids as well, and the resistance to these fatty acids was shown to be enhanced by inactivation of the *aas* gene. On the basis of these observations, we developed an efficient method to isolate *aas*-deficient mutants from cultures of *Synechocystis* cells by counter selection using linoleic acid or linolenic acid as the selective agent. A variety of *aas* mutations were found in about 70 % of the FFA-resistant mutants thus selected. Various *aas* mutants were isolated also from *Synechococcus* sp. PCC 7002, using lauric acid as a selective agent. Selection using FFAs was useful also for construction of markerless *aas* knockout mutants from *Synechocystis* sp. PCC 6803 and *Synechococcus* sp. PCC 7002. Thus, genetic engineering of FFA-producing cyanobacterial strains would be greatly facilitated by the use of the FFAs for counter selection.

**Electronic supplementary material:**

The online version of this article (doi:10.1007/s00253-016-7850-8) contains supplementary material, which is available to authorized users.

## Introduction

Acyl-acyl carrier protein synthetase (Aas), which is found in most organisms performing oxygenic photosynthesis, catalyzes esterification of free fatty acids (FFAs) to acyl carrier protein (ACP). In cyanobacteria, Aas mediates incorporation of exogenously added FFAs into membrane lipids, but its natural substrate is thought to be the FFAs produced in the cell, because *aas-*deficient mutants secrete FFAs into the medium (Kaczmarzyk and Fulda 2010). Cyanobacterial cells produce FFAs by deacylation of the membrane lipids (Kaczmarzyk and Fulda 2010; Takatani et al. 2015). Since deficiency of Aas results in destabilization of photosystem II and renders the cells sensitive to high light (>400 μE m^−2^ s^−1^), the Aas-mediated recycling of FFAs is deduced to be important for acclimation of cyanobacteria to highlight conditions (Takatani et al. 2015). The function of Aas, however, can be detrimental to cyanobacterial cells; Exogenously added polyunsaturated fatty acids, i.e., linoleic acid (18:2) and α-linolenic acid (18:3) exert toxic effects to the cells (Sakamoto et al. 1998; Maeda et al. 2005), and the toxicity of 18:3 was shown to be dependent on Aas in *Synechocystis* sp. strain PCC 6803 (von Berlepsch et al. 2012). It is supposed that the concentration gradient of the FFA, which is maintained by 18:3-ACP formation on the cytoplasmic side of the plasma membrane, is responsible for the uptake of the toxic FFA (von Berlepsch et al. 2012). Although the loss of Aas should result in accumulation of endogenously produced FFAs, their toxicity seems to be insignificant as compared with that caused by the uptake of exogenously added 18:3 at least under the light conditions commonly used in the laboratories (30–140 μE m^−2^ s^−1^).

To develop a reliable method for photosynthetic biofuel production, attempts have been made to increase FFA productivity of genetically engineered cyanobacteria (Liu et al. 2011b; Ruffing 2014; Kato et al. 2015). Targeted inactivation of *aas* is an essential step of construction of the FFA-producing cyanobacterial strains. Combined with expression of foreign thioesterase gene(s), the loss of FFA recycling allows for production of large amounts of FFAs (Liu et al. 2011b; Ruffing and Jones 2012), but further increase of FFA production requires various additional gene manipulations aimed at overexpression of Rubisco and the enzymes of the FFA biosynthesis pathway, inactivation of the PHB biosynthesis pathway, modification of the peptidoglycan layer, overexpression of a FFA exporter, etc. (Liu et al. 2011a; Ruffing and Jones 2012; Ruffing 2013; Ruffing 2014; Kato et al. 2015; Kato et al. 2016). Multistep transformation of cyanobacterial cells is time consuming and further complicated by the limited number of available antibiotic resistance markers. Simple methods to introduce relevant mutations and genes are needed. In this study, we developed a simple method to isolate *aas*-deficient mutants from wild-type (WT) population of *Synechocystis* sp. PCC 6803 and *Synechococcus* sp. PCC 7002 cells using FFAs as the selective agents. Counter selection is shown to be effective also for targeted inactivation of *aas* without using a marker gene. Potential usefulness of counter selection in markerless integration of the genes required to increase the FFA productivity is discussed.

## Materials and methods

### Organisms and culture conditions

Cells of *Synechocystis* sp. PCC 6803 and *Synechococcus* sp. PCC 7002 were grown at 30 °C under continuous illumination at 50 μE m^−2^ s^−1^ provided by fluorescent lamps using nitrate as the nitrogen source. The liquid medium used was a modification of BG11 (Stanier et al. 1971) described previously (Suzuki et al. 1995). Solid medium was prepared by addition of 1.5 % (*w*/*v*) agar and 0.3 % (*w*/*v*) sodium thiosulfate to the liquid medium. For growth of *Synechococcus* sp. PCC 7002, the media were supplemented with 0.25 M of NaCl and 0.01 μg/ml of vitamin B_12_. Liquid cultures were bubbled with air supplemented with 2 % (*v*/*v*) CO_2_, and the incubation of cells on the agar plate was in the air. When appropriate, kanamycin was added to the medium at 15 μg ml^−1^.

### Targeted inactivation of *aas* using a drug resistance marker

The nucleotide sequences of the *aas* genes encoding acyl-carrier protein synthetase from the two species of cyanobacteria were obtained from the CyanoBase website (http://genome.microbedb.jp/cyanobase/). The *aas* genes of *Synechocystis* sp. PCC 6803 and *Synechococcus* sp. PCC 7002 were amplified by PCR using the KOD plus DNA polymerase (Toyobo, Osaka, Japan) using the primer pairs a1/a6 and b2/b8, respectively (Table 1; Fig. S1). The amplified DNA fragments were cloned into the pGEM-T easy vector (Promega, Madison, WI, USA). To inactivate the *aas* genes in cyanobacteria, a 3.8 kb DNA fragment carrying the *sacB* gene and the kanamycin resistance gene was excised from the plasmid pRL278 (accession number in GeneBank L05083) and ligated into the *Sma*I and the *Bgl*II recognition sites in the *aas* genes cloned from *Synechocystis* sp. PCC 6803 and *Synechococcus* sp. PCC 7002, respectively. The resultant plasmids were used to transform the wild-type cells through homologous recombination into kanamycin resistance. After three rounds of streak purification of single colonies, absence of the wild-type *aas* copy in selected colonies was confirmed by PCR. The *aas*-deficient *kan* ^r^ mutants thus obtained were named dAS11 and dAS21 for *Synechocystis* sp. PCC 6803 and *Synechococcus* sp. PCC 7002, respectively (Fig. S1).

**Table 1:** Oligonucleotides used for PCR in this study

Strain	DNA target	Primer	Sequence (5′-3′)	Position of 5′ end	Direction
*Synechocystis* sp. PCC 6803	s1r1609	al	ACGCTTTGGTGATGAACACTGG	−198	Forward
		a2	CTGGGCTATCACCGGAGAAAAT	+228	Forward
		a3	AAGGGGTGATGCTCAGCCACGG	+761	Forward
		a4	AGCCGGGGCACACCGACAATGTG	+998	Reverse
		a5	GGATTTGCCCCCCGAAACCCAAGG	+1493	Forward
		a6	CCCATAGGCCTTAGATCGTGTTTG	+2214	Reverse
*Synechococcus* sp. PCC 7002	SYNPCC7002_A0675	b1	AGGGCCATGAGTTCGGCGTTGACT	−962	Forward
		b2	TCAGAAGATCCCGACCCTTG	−235	Forward
		b3	AGATTCCGCCATTCGGATCCCGGGCAAGCCGAAATCATGGCTAC	+3	Reverse
		b4	ACTTTTCCCAACTGATGACCCTCG	+494	Forward
		b5	CCATGTAACCGGGCTTGTAGGTTTG	+826	Reverse
		b6	CCTCGGCAACAAACTCGTTTACG	+1053	Forward
		b7	GTAGCCATGATTTCGGCTTGCCCGGGATCCGAATGGCGGAATCT	+1947	Forward
		b8	AGATTCCGCCATTCGGATCG	+1965	Reverse
		b9	CCTTTCACTGAGGCCACATC	+2879	Reverse

### Effects of FFAs on growth of cyanobacteria

For viability assays on the agar plates, cultures in the late logarithmic phase of growth were diluted to an optical density of 0.5 at 730 nm, and then serially diluted with fresh BG-11 medium. A 5 μl aliquot from each dilution was spotted onto BG-11 plates containing 0.1 mM of different fatty acids. The plates were incubated for 1 week at 30 °C under illumination at a light intensity of 20 μE m^−2^ s^−1^.

## Results

### Effects of Aas deficiency on cellular sensitivity to various FFAs

**Fig. 1:**
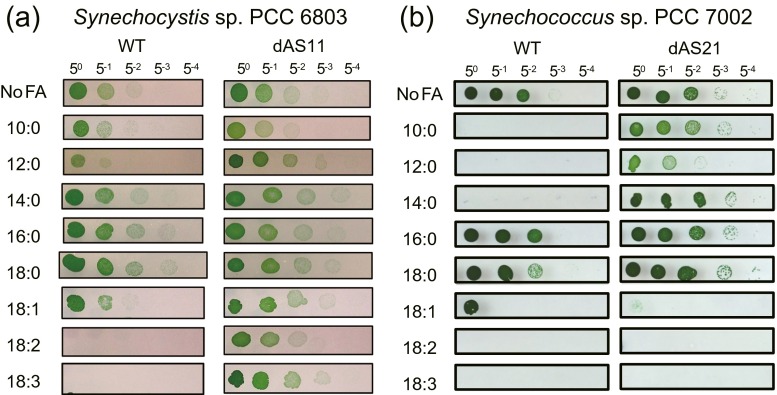
Effects of various FFAs on growth of the wild-type strain and the *aas*-deficient mutant of *Synechocystis* sp. PCC 6803 (**a**) and *Synechococcus* sp. PCC7002 (**b**). Cells grown in liquid medium to the late logarithmic phase of growth were diluted with fresh liquid medium to give an optical density of 0.1 at 730 nm. Five microliters of the cell suspension and its 5-fold serial dilutions were spotted on solid media supplemented with 100 μM of the FFAs and grown for 7 days under illumination at 20 μE m^−2^ s^−1^. *Numbers* on the *top* indicate the dilution factor. Results from one of the three experiments, which yielded essentially the same results, are shown

Figure 1a shows the effects of various FFAs on growth of the WT and the *aas*-deficient mutant strains of *Synechocystis* sp. PCC 6803. As previously reported, the WT *Synechocystis* cells grew fine in the presence of palmitic acid (16:0) and stearic acid (18:0) (Ruffing and Trahan 2014). The cells grew well also in the presence of myristic acid (14:0). In accordance with the previously published results (Sakamoto et al. 1998), the WT cells were sensitive to the C_18_ polyunsaturated FFAs, i.e., linoleic acid (18:2) and linolenic acid (18:3), while they could tolerate the presence of the monounsaturated FFA, oleic acid (18:1). The engineered *aas*-deficient mutant dAS11 was resistant not only to 18:3 as previously reported (von Berlepsch et al. 2012) but also to 18:2. The mutant was somewhat more tolerant to lauric acid (12:0) than WT.

Figure 1b shows the effects of FFAs on growth of the WT and the *aas*-deficient mutant strain of *Synechococcus* sp. PCC 7002, a model cyanobacterium that is thought to be useful for FFA production under the outdoor conditions because of its tolerance to strong irradiance and high salt concentrations (Reed and Stewart 1985; Ludwig and Bryant 2012). Similar to the results obtained with *Synechocystis* sp. PCC 6803, and in accordance with the previously reported results, the WT cells of *Synechococcus* sp. PCC 7002 grew well in the presence of 16:0 and 18:0 (Ruffing and Trahan 2014) but failed to grow in the presence of 18:2 and 18:3 (Sakamoto et al. 1998; Ruffing and Trahan 2014) (Fig. 1b). *Synechococcus* sp. PCC 7002 did not grow at all in the presence of 100 μM of 10:0, 12:0, and 14:0, but the engineered *aas* mutant dAS21 was resistant to these fatty acids. Unlike in *Synechocystis* sp. PCC 6803, deficiency of Aas did not confer the cells the resistance to the long-chain polyunsaturated fatty acids 18:2 and 18:3.

### Isolation of *aas* mutants from WT cell population using FFAs as the selective agents

**Fig. 2:**
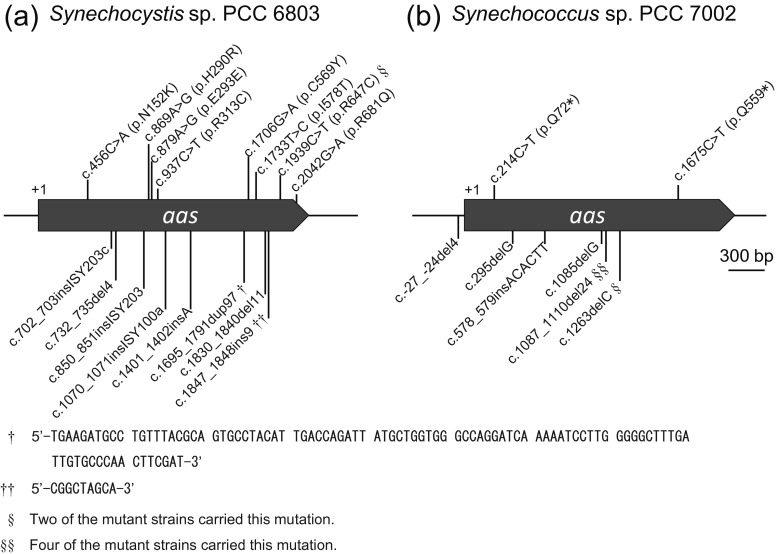
Mutations found in the *aas* gene of the 18:2- or 18:3-resistant mutant strains of *Synechocystis* sp. PCC 6803 (**a**) and 12:0-resistant mutant strains of *Synechococcus* sp. PCC7002 (**b**). Nucleotide substitutions are shown above the maps, together with the resulting amino acid substitutions. Insertions and deletions are shown below the maps. *c* coding region, *p* protein, *del* deletion, *dup* duplication, *ins* insertion. The sequences of the 97-base duplication (dup97; *dagger*) and the 9-base insertion (9ins; *double dagger*) are shown below the map in **a**

Although the WT cells of *Synechocystis* sp. PCC 6803 seemed to have completely died in the presence of 100 μM of 18:2 or 18:3 in 7 days, two and one colonies came up in the spot of undiluted cell suspension on the 18:2- and 18:3-containing agar plates, respectively, after 14 days of incubation. Transfer of the cells to new 18:2- and 18:3-containing media showed that they were resistant to both of the FFAs (not shown). PCR amplification and sequencing of the *aas* locus of these clones showed that they have mutations in the *aas* gene. These results indicated that *aas*-deficient mutants can be readily identified and isolated by using 18:2 or 18:3 as the selecting agent. For larger scale screening of naturally occurring mutations of the *aas* gene, WT *Synechocystis* cell suspension (OD_730_ = 3) was diluted to OD_730_ = 0.5 by BG11 and spread 100 μL of cell suspension onto agar plates containing 100 μM of 18:2 and 18:3, respectively. After 14 days of incubation, 12 and 54 colonies appeared on the 18:2- and 18:3-containing media, respectively. Cells from these colonies were subcultured for three passages on the FFA-containing media. DNA was subsequently purified from 10 and 17 of the lines obtained from the 18:2- and 18:3-containing media, respectively and subjected to PCR amplification and sequence analysis of the *aas* locus. Of the 27 strains examined, 17 strains carried a mutation in *aas*. There were 16 mutant alleles for *aa*s, each carrying an indel or a base substitution that results in a frame shift, an amino acid substitution, or no amino acid substitution in the encoded protein (Fig. 2a).

The *aas*-deficient *Synechococcus* sp. PCC 7002 mutant failed to grow in the presence of 18:2 or 18:3, but it could grow in the presence of 10:0, 12:0, and 14:0, suggesting that these FFAs may be used as the selecting agents to identify and isolate *aas*-deficient mutants from the wild-type population of the cells. To determine whether 12:0 can be used for selection of *aas*-deficient mutants from the WT cultures of *Synechococcus* sp. PCC 7002, 100 μl of the cell suspension (OD_730_ = 0.5) was spread onto agar plates containing 100 μM of 12:0. Seventeen colonies obtained after 14 days of incubation were subcultured for three passages on the 12:0-containing medium. PCR amplification and sequence analysis of the *aas* locus revealed that 12 of the 17 strains carried a mutation in *aas*. There were eight mutant alleles for *aa*s, each carrying an indel or a base substitution that would result in a frame shift or an amino acid substitution (Fig. 2b).

### Markerless knockout of the *aas* gene using FFAs as the selective agents

**Fig. 3:**
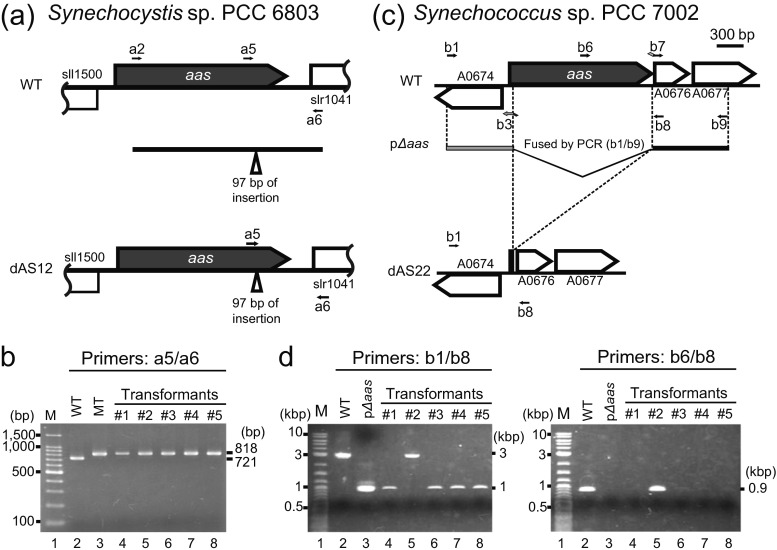
Construction of markerless *aas* mutants of *Synechocystis* sp. PCC 6803 (**a**, **b**) and *Synechococcus* sp. PCC 7002 (**c**, **d**) by one-step transformation, using 18:3 and 12:0 as selective agents, respectively. **a** Diagram showing the map of the *aas* locus of wild-type *Synechocystis* sp. PCC 6803 (WT), the DNA fragment used for the mutagenesis, and the map of the *aas* locus of the resultant mutant (dAS12). The primers used for PCR are also shown. **b** DNA fragments amplified from five lines of the 18:3-resistant transformants by PCR using the primer pair a5/a6. The PCR products were analyzed by electrophoresis on a 2 % agarose gel. Lane *1*, molecular size markers; lanes *2* and *3*, PCR products amplified from WT and the mutant carrying a 97-bp insertion in *aas*, respectively; lanes *4*–*8*, PCR products amplified from the selected transformants. **c** Diagram showing the map of the *aas* locus of wild-type *Synechococcus* sp. PCC 7002, the DNA fragment cloned into the p∆*aas* plasmid to be used for the mutagenesis, and the map of the *aas* locus of the resultant mutant (dAS22). **d** DNA fragments amplified from five lines of the 12:0-resistant transformants by PCR using the primer pairs b1/b8 and b6/b8. The PCR products were analyzed by electrophoresis on a 0.8 % agarose gel. Lane *1*, molecular size markers; lanes *2* and *3*, PCR products amplified from the WT genome and the p∆*aas* plasmid, respectively; lanes *4*–*8*, PCR products amplified from the selected transformants

Since FFAs were successfully used as selective agents for identification and isolation of the cells bearing spontaneous mutations in the *aas* gene, we further tried targeted inactivation of *aas* by counter selection, without using an antibiotic resistance marker. One of the *Synechocystis aas* mutants obtained by screening of 18:3-tolerant cells had a 97-base insertion in the *aas* ORF (Fig. 2a). A 2084-bp DNA fragment of the *aas* gene, carrying the 97-bp insertion, was amplified from the mutant by PCR using the primer pair a2/a6 (Fig. 3a) and used to transform WT *Synechocystis* sp. PCC 6803 cells to 18:3 resistance. Colonies from five independent transformant lines were isolated after three serial streak purifications on 18:3-containing medium, and all of them were shown to carry the 97-base insertion in the *aas* gene by PCR analysis (Fig. 3b) and nucleotide sequence analysis (not shown). Markerless knockout of the *aas* gene was attempted also in *Synechococcus* sp. PCC 7002, using 12:0 as the selective agent for counter selection. To this end, a 965-bp DNA fragment carrying the initiation codon of *aas* and 962 bases of its 5′-flanking sequence was amplified by PCR using the primers b1 and b3 (Fig. 3c). A 933-bp DNA fragment of the 3′-flanking sequence of *aas*, carrying the bases +3 to +935 with respect to the *aas* termination codon, was also amplified by PCR using the primers b8 and b9 (Fig. 3c). The two fragments were jointly cloned into the pGEM-Teasy plasmid to construct the plasmid p*∆aas* (Fig. 3c). The plasmid was used to transform WT cells of *Synechococcus* sp. PCC 7002 to 12:0 resistance, and colonies of five independent 12:0-resistant lines were streak purified. PCR analysis showed that the *aas* ORF had been deleted from the genome of four out of the five transformants (Fig. 3d). The remaining one transformant (Fig. 3d, #2) carried the wild-type *aas* gene, indicating that it had acquired the tolerance to 12:0 by a mutation(s) located elsewhere on the genome. Taken together, nine out of the ten FFA-tolerant transformants obtained from *Synechocystis* sp. PCC 6803 and *Synechococcus* sp. PCC 7002 were defective in the *aas* gene. Although cyanobacteria have multiple copies of genomic DNA and are notorious for the difficulties in segregation of mutant and WT chromosomes, there was no sign of wild-type *aas* in the nine transformants (Fig. 3b, d). These results demonstrated that FFA-based counter selection applies strong enough selection pressure on cyanobacterial cells, allowing for inactivation of *aas* without using a drug resistance marker.

## Discussion

Cyanobacteria contain C_16_ and C_18_ fatty acids as the acyl moiety of their membrane lipids. They are resistant to high concentrations (500 μM) of exogenously added 16:0 and 18:0 (Ruffing and Trahan 2014). Unlike the long-chain saturated fatty acids, however, exogenous long-chain polyunsaturated fatty acids, i.e., 18:2 and 18:3 are toxic to cyanobacteria (Sakamoto et al. 1998; von Berlepsch et al. 2012; Ruffing and Trahan 2014). In the present results, 18:1 was also toxic to *Synechococcus* sp. PCC 7002 (Fig. 1b), although cyanobacteria have been reported to be generally resistant to 18:1 (Sakamoto et al. 1998; Kato et al. 2015). Remarkably, the toxic effect of these FFAs was not abolished by *aas* inactivation in *Synechococcus* sp. PCC 7002. This contrasts with the results obtained in *Synechocystis* sp. PCC 6803, where the tolerance to 18:2 and 18:3 is acquired by inactivation of Aas (von Berlepsch et al. 2012) (Fig. 1a). Since Aas is presumed to mediate the toxicity of the exogenous FFAs by facilitating their entry into the cells (von Berlepsch et al. 2012), the sensitivity of the *aas*-deficient *Synechococcus* sp. PCC 7002 cells to the unsaturated FFAs may suggest the presence of an *aas*-independent mechanism for uptake of unsaturated FFAs. The presumed uptake mechanism is unlikely to mediate the uptake of 10:0, 12:0, and 14:0, because the *aas*-deficient mutant of *Synechococcus* sp. PCC 7002 is tolerant to these FFAs (Fig. 1b).

It should be noted that the sensitivity of cyanobacterial cells to externally added FFAs is determined not only by the cellular capacity of FFA uptake but also by the activity of FFA export out of the cell. In *Synechococcus elongatus* PCC 7942, inactivation of the genes coding for an RND-type export system abolishes the ability of the cells to grow on solid media containing 10:0 (100 μM), 12:0 (25 μM), 14:0 (500 μM), 18:1 (200 μM), and 18:3 (25 μM), respectively (Kato et al. 2015). The sensitivity of WT *Synechococcus* sp. PCC 7002 cells to all the FFAs tested, excluding 16:0 and 18:0 (Fig. 1b), may suggest the absence of an effective FFA export system in this strain.

Although many of the FFA-resistant mutants isolated from *Synechocystis* sp. PCC 6803 and *Synechococcus* sp. PCC 7002 carried a mutation in the *aas* gene (Fig. 2), there were mutants carrying the wild-type *aas* gene. There also was an 18:3-tolerant strain carrying a silent mutation in *aas* (Fig. 2a, c.879A>G), which is likely to carry a mutation in another locus on the genome. The mutations responsible for the FFA tolerance of these strains may include those affecting the uptake and export of FFAs, but they may also include mutations that lead to better protection of cellular activities against the toxic effects of FFAs in the cell. It has been shown that cyanobacterial mutants producing large amounts of FFAs suffer from toxicity of the products accumulating in the cell (Kaczmarzyk and Fulda 2010; Kato et al. 2016). Any mutation that mitigates the toxic effects of the FFAs in the cell would be useful for enhancement of growth and productivity of FFA-producing cyanobacterial mutants. Genome re-sequencing analysis of the FFA-resistant mutants is being performed to identify the mutations that could enhance tolerance of the cell to intracellular accumulation of FFAs.

The insertional interruption of the *aas* gene without using an antibiotic resistance marker (Fig. 3a, c) is of particular relevance with construction of FFA-producing mutants from cyanobacteria, because markerless integration of potentially useful gene(s) and inactivation of *aas* can be achieved by a single transformation step. This would greatly reduce the time and resource required for construction of FFA producing strains. The selection of naturally occurring mutations of *aas* using toxic FFAs as the selection agents (Fig. 2), on the other hand, would enable isolation of FFA-secreting mutants from non-transformable cyanobacterial species, e.g., the *Arthrospira* species.

Begemann et al. (2013) recently reported a counter selection system in *Synechococcus* sp. PCC 7002, using acrylate as the selective agent and *acsA* as the target gene. The product of *acsA* is an acetate-CoA ligase, whose functioning with acrylate as the substrate is toxic. Counter selection of *acsA* was shown to be useful not only for isolation of spontaneous *acsA* mutants or targeted inactivation of *acsA* but also for markerless integration of a foreign gene in exchange with *acsA*. Since a gene cassette carrying *acsA* and an antibiotic resistance marker can be targeted to a desired locus on the genome, it is possible to remove a defined region from the genome or to replace a genomic region with a foreign gene via marker exchange-eviction (Begemann et al. 2013). Because deficiency of Aas results in destabilization of PSII, rendering the cells sensitive to high-intensity light (Takatani et al. 2015), *aas* may be used not only for counter selection but also for positive selection. This would enable development of a simple method for modification of the genomic DNA via integration and/or removal of defined genetic elements without using antibiotics.

## Electronic supplementary material

Fig. S1 (PDF 238 kb)
