# Supplementary material for: A simple method for isolation and construction of markerless cyanobacterial mutants defective in acyl-acyl carrier protein synthetase
Source: Appl Microbiol Biotechnol. 2016 Oct 4;100(23):10107–13. doi: 10.1007/s00253-016-7850-8 (PMC5102962; doi:10.1007/s00253-016-7850-8)
Supplement: Supplementary file 1 — (PDF 238 kb) [file 253_2016_7850_MOESM1_ESM.pdf]

Supplementary material

Applied Microbiology and Biotechnology

A simple method for isolation and construction of markerless cyanobacterial mutants defective in acyl-acyl carrier protein synthetase

Kouji KOJIMA[1,4,5], Sumie KETA[1,4], Kazuma UESAKA[2,4], Akihiro KATO[2,4], Nobuyuki TAKATANI[2,4], Kunio IHARA[3,4], Tatsuo OMATA[2,4], Makiko AICHI1[1,4,†]

[1]Department of Biological Chemistry, Chubu University, Kasugai, 487-8501 Japan

[2]Graduate School of Bioagricultural Sciences, Nagoya University, Nagoya, 464-8601 Japan

[3]Center for Gene Research, Nagoya University, Nagoya, 464-8602 Japan

[4]Japan Science and Technology Agency, CREST

[5]Present address: Graduate School of Bioagricultural Sciences, Nagoya University, Nagoya, 464-8601 Japan.

†To whom correspondence should be addressed

E-mail: makiko@isc.chubu.ac.jp

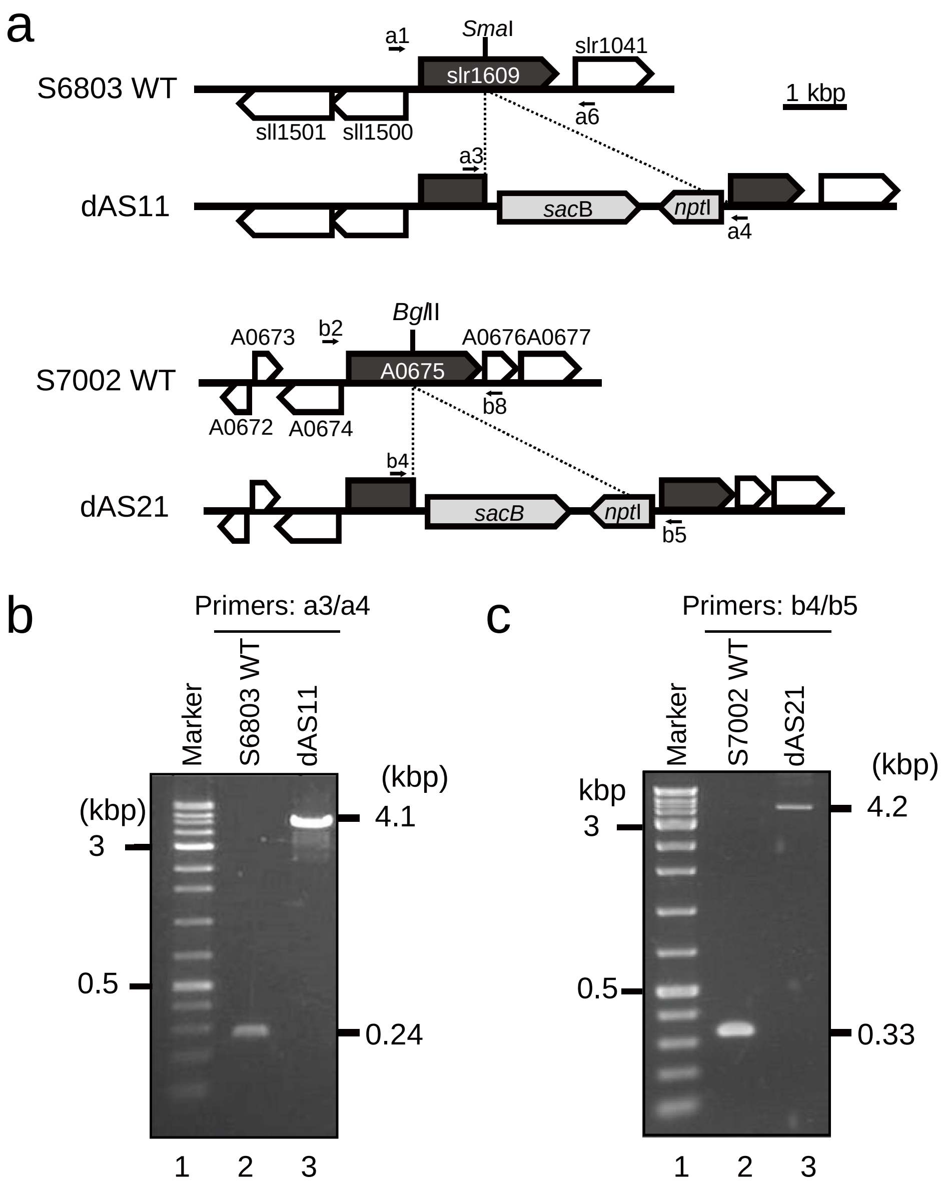

Figure S1. Construction of *aas* insertional mutants from *Synechocystis* sp. PCC 6803 and *Synechococcus* sp. PCC 7002. a Diagrams showing the maps of the *aas* loci of the wild-type and the mutant of the two cyanobacterial strains. The PCR primers used to screen for homozygous strains in the *aas* locus are also shown. The primer pair a3/a4 amplifies 0.24-kb and 4.1-kb DNA fragments from *Synechocystis* sp. PCC 6803 WT and the dAS11 mutant, respectively. The primer pair b4/b5 amplifies 0.33-kb and 4.2-kb fragments from *Synechococcus* sp. PCC 7002 WT and the dAS21 mutant, respectively. **b** and **c** DNA fragments amplified from the dAS11 and dAS21 mutants using the primer pairs a3/a4 and b4/b5, respectively. The PCR products were analyzed by electrophoresis on a 0.8% agarose gel.
